# Flexible decision-making in grooming partner choice in sooty mangabeys and chimpanzees

**DOI:** 10.1098/rsos.172143

**Published:** 2018-07-11

**Authors:** Alexander Mielke, Anna Preis, Liran Samuni, Jan F. Gogarten, Roman M. Wittig, Catherine Crockford

**Affiliations:** 1 Department of Primatology, Max Planck Institute for Evolutionary Anthropology, Leipzig, Germany; 2 Centre Suisse de Recherches Scientifiques en Côte d'Ivoire, Taï Chimpanzee Project, Abidjan, Côte d'Ivoire; 3 Department of Biology, McGill University, Montreal, Canada; 4 P3: ‘Epidemiology of Highly Pathogenic Microorganisms’, Robert Koch Institute, Berlin, Germany

**Keywords:** grooming, bystanders, sooty mangabey, chimpanzee, decision-making

## Abstract

Living in permanent social groups forces animals to make decisions about when, how and with whom to interact, requiring decisions to be made that integrate multiple sources of information. Changing social environments can influence this decision-making process by constraining choice or altering the likelihood of a positive outcome. Here, we conceptualized grooming as a choice situation where an individual chooses one of a number of potential partners. Studying two wild populations of sympatric primate species, sooty mangabeys (*Cercocebus atys atys*) and western chimpanzees (*Pan troglodytes verus*), we tested what properties of potential partners influenced grooming decisions, including their relative value based on available alternatives and the social relationships of potential partners with bystanders who could observe the outcome of the decision. Across 1529 decision events, multiple partner attributes (e.g. dominance ranks, social relationship quality, reproductive state, partner sex) influenced choice. Individuals preferred to initiate grooming with partners of similar global rank, but this effect was driven by a bias towards partners with a high rank compared to other locally available options. Individuals also avoided grooming partners who had strong social relationships with at least one bystander. Results indicated flexible decision-making in grooming interactions in both species, based on a partner's value given the local social environment. Viewing partner choice as a value-based decision-making process allows researchers to compare how different species solve similar social problems.

## 1. Introduction

Animals living in social groups engage in competitive and cooperative social interactions with other group members. To balance short- and long-term costs and benefits, animals have to decide when and how to interact with conspecifics so that the choice yields higher profit than an interaction with a random partner [1]. Increasing evidence suggests that social animals exchange cooperative acts (i.e. all interactions that, on average, result in a net gain for all participants [2]) as services in a social market where partners differ in the commodities they can offer as a return [3]. The value of services and commodities follows the market forces of supply and demand [4]. An individual can increase the benefits it acquires from cooperating by selecting a cooperation partner that can reciprocate specific services the individual needs (partner choice [3–5]), and afterwards ensuring reciprocation of the service over repeated cooperative events (partner control [6–8]). In a social market, mechanisms that improve partner choice should be selected for [7]. Partner choice might be optimized if individuals can accurately evaluate the relative benefits of investing in one partner over another in a particular social context. However, the assessment and comparison of partner values can be complex when integrating information about diverse partner characteristics and commodities partners might offer [9]. Each potential choice is associated with specific benefits (e.g. access to resources partner can offer [5,10]), costs (e.g. time invested by the donor [11]) and risks (e.g. likelihood partner does not reciprocate [12]), which are in constant flux due to changing ecological and social environment [13,14]. This suggests that flexibly adapting their decision-making process based on changing social and ecological conditions might enable animals to maximize fitness benefits from choosing a cooperation partner.

Primate grooming has been interpreted as an important commodity that can be exchanged for other services [15–17]. Groomers can benefit immediately (e.g. by eliciting reciprocated grooming that reduces physiological stress [18,19] and ectoparasite load [20]; by gaining access to infants [21–23]) or with a delay (e.g. by eliciting coalitionary support [24–27], group defence [28–30], reducing the likelihood of aggression [31] or increasing access to food [15,32–34] and mating [14,35]). Partner value can also change due to demographic or ecological changes in the group (e.g. rank changes, migration events, food availability [10,36], death of group members [37], birth [23]). Some primate species have flexible association patterns and exhibit fission–fusion dynamics (with subgroups varying in composition and duration [38]). Primates living in fission–fusion societies need to make grooming decisions in the face of frequently changing conditions regarding the relative value of potential partners and relationships of potential partners with others [13].

Primate grooming interactions thus represent an ideal opportunity for examining the flexibility of the decision-making process underlying partner choice. Market theories predict that individuals adapt their partner choice to the local social environment which determines the supply [3,14]; for example, male long-tailed macaques invest less into grooming particular females when more other females are available [14]. Partner choice in grooming has often been studied using across-dyad correlational approaches aggregating data over longer time periods [8,39,40]. Such approaches are limited in their ability to establish the choices responsible for observed distributions [8,39,40]. Here, we look at partner choice as a decision-making event, where an individual has multiple potential partners before deciding to groom one of them. At the same time, all potential partners who were not chosen witness the choice as bystanders. This approach allows us to incorporate variables that are too fleeting to be tested in correlational approaches, such as the reproductive state of potential partners, or that usually have to be represented statically despite being dynamic, such as social relationships. Given that grooming as a service is exchanged for a number of commodities, partners vary along a large number of characteristics (e.g. dominance rank, sex, past interactions, reproductive state) that can potentially influence this decision-making process. The comparative assessment of multiple commodities has been argued to be a particularly complex task for animals [41,42].

In grooming, bystanders can impact the outcome of interactions by actively disrupting or joining grooming [43], or by inciting partner switching by either of the groomers [44]. If the goal of partner choice in grooming is to maximize the time one has with a partner, then groomers should not invest in targets who are more likely to switch partners or who might attract interventions by others [12]. Partner switching of the groomee could be more likely if attractive bystanders are around, leading for example chimpanzees to invest less in a grooming bout if high-ranking bystanders are present [12,44]. To avoid interventions, an individual should refrain from grooming a partner who has strong social relationships with bystanders [43]. In this context, primates could exhibit inhibitory control [38,45], by choosing an alternative if access to the preferred partner is limited by the social environment.

The presence and absence of group members can influence grooming interactions by changing the relative value of a commodity a partner has to offer [14,36]. Previous studies have represented grooming partners' rank and social relationships in comparison to everyone in the community (e.g. [46,47]). In these models, every group member is represented by one value per dimension (e.g. rank). However, when association patterns are fluid, especially in species with high fission–fusion dynamics, a limited availability of grooming partners at any given moment might mean that partner values are not fixed [38]. For example, an individual's relative rank could fluctuate depending on the presence of higher-ranking group members. Here, we test whether groomers evaluate potential partners' rank and social relationship quality based on their global value or a more fluid relative value that represents their worth compared to other options available. To test which factors influence grooming decisions in different social systems, we collected data in two primate species living sympatrically in Taï National Park, Cote d'Ivoire.

Sooty mangabeys (*Cercocebus atys atys*) and western chimpanzees (*Pan troglodytes verus*) both live in large multi-male, multi-female societies, but with social systems that should differ in terms of the impact of the social environment on cooperative interactions. Sooty mangabeys are female philopatric, probably matrilineal [48,49], and exhibit strong contest competition [49], leading to a despotic dominance system where rank is highly predictive of outcomes in competitive situations [43,49]. In such a social system, theory predicts a preference for grooming dominants and kin [5,50,51]. Previous studies in mangabeys offer partial support for this, showing attraction towards closely ranked group members, more grooming investment by low-ranking partners [52], a preference for kin [48], as well as for mothers of young infants [23]. Similar to what has been observed in chacma baboon males [53], adult mangabey males do not groom each other [43]. Mangabeys exhibit weak fission–fusion dynamics: the entire group usually travels in the same direction, dispersed over a distance of 50–150 m, but without clearly separated subgroups (A. Mielke 2015, personal observation). Despite their proximity, not all group members are in visual contact at any given moment, due to the dense rainforest environment, meaning that the immediate social environment in which grooming occurs is still variable [43]. In a previous study, we showed that mangabeys monitor grooming of other group members and intervene adaptively [43], indicating that mangabeys possess triadic awareness for the ranks and relationships of others. Based on the available information, we predicted that mangabeys make multidimensional grooming decisions by comparing multiple properties of potential partners, such as their social relationship quality, sex, reproductive state, and that dominance ranks are a particularly strong factor. As no strong fission–fusion dynamics exist, the cognitive mechanisms for tracking changes in the immediate social environment may be limited [13,45]. Thus, we predicted, first, that the relationships between potential partners and bystanders would not play a significant role in partner choice. Second, we predicted that individuals would choose grooming partners that were close to them in global rank or were globally high-ranking [52], independent of the composition of bystanders. This would result in the relative rank model showing the same effects as the global rank model, as each individual in the community has a value based on their global rank and this value determines whether they are groomed or not.

Chimpanzees are male philopatric [54]. In some chimpanzee communities, males display a grooming bias towards either high-ranking or closely ranked group members [55–57], while in others they do not [58,59], which has been interpreted to be a product of differences in the steepness of dominance hierarchies between communities [55]. The chimpanzee communities studied here exhibited moderate levels of despotism during the time period of this study [43]. Female chimpanzees in Taï are more gregarious than at many other field sites [60] and focus their grooming on a small number of preferred partners without a clear bias towards high-ranking individuals [61]. Chimpanzees have high levels of fission–fusion dynamics, creating clearly divided subgroups that can persist for hours or days [62], resulting in changing social environments in which grooming decisions are made. We predicted that chimpanzees will differ from mangabeys by exhibiting a reduced emphasis on rank as a choice parameter. Furthermore, we predicted that in chimpanzees, where fission–fusion dynamics create a more dynamic social environment, the value of grooming partners of specific dominance rank will not be fixed but will be dependent on the availability of other high-ranking partners. For example, the likelihood that the individual with the third rank globally is chosen as grooming partner is dependent on the local presence of the higher-ranking alternatives. This would create a mismatch between the impact of global and relative rank in the analysis. Finally, we predicted that if potential partners have close affiliation partners available, they will be less likely to be selected as a grooming partner, as this presence might impede the successful outcome of the grooming interaction.

## 2. Methods

### 2.1. Data collection

Grooming data were collected in Taï National Park, Côte d'Ivoire [54] from 2013 to 2015, using half- and full-day continuous focal animal sampling. All individuals were well habituated to human presence, allowing close-range observation from around 7–10 m distance. Three observers (A.M., A.P., L.S.; inter-observer reliability greater than 90%) recorded all social interactions of adult male and female chimpanzees (above 12 years of age) in the ‘South’ (A.M., A.P., L.S.) and ‘East’ (A.P., L.S.) communities and adult (above 5 years) sooty mangabeys (A.M.; table 1). Subadult group members in all communities and adults with insufficient focal data in the two chimpanzee communities (East: two adult females; South: five adult females) were removed from the dataset, both as potential partners and when computing the bystander variable and relative ranks, as we could not reliably determine their ranks and social relationships. Data were collected using customized CyberTracker data collection software (CyberTracker Conservation 2013). In chimpanzees, individuals that were within the visual range (usually 30–50 m) of the focal individual were recorded continuously as members of the party and constituted the bystanders of a grooming bout [43,61]. For mangabeys, where no clear-cut parties exist, we recorded all individuals that appeared in visual range during a 5-minute period, and considered these the bystanders for grooming of the focal in this time period [43].

**Table 1.** Characteristics of the study groups, observation time, grooming interactions and unique compositions of bystanders in which decisions were made.

	focal individuals		potential partners		observation hours		grooming initiations		grooming initiations per hour		
	male	female	male	female	f	m	f	m	f	m	unique party compositions
mangabey	0	12	4	20	502 h	128 h	157	0	0.31	0	156
chimpanzee East	5	4	5	11	505 h	1831 h	100	540	0.20	0.30	438
chimpanzee South	5	6	5	7	894 h	2088 h	79	653	0.09	0.31	451

To capture the grooming partner choice of individuals, we analysed only grooming events in which the focal individual was the first individual to groom another, as these are potentially the only ones where the focal individual's behaviour is not a response to the choice of the potential partner [44]. We did not consider grooming invitation gestures, even though they exist in both species, as they were not systematically collected. We grouped grooming interactions with other adult group members into grooming sessions, i.e. all consecutive grooming interactions of the focal with any adult group member separated by less than 5 min. Only the first choice of each session was retained, as partner choice of later partners might not be independent of the first choice. For each choice, all adult individuals that were present when grooming was initiated formed the set of potential partners; for each potential partner, all other adult individuals who were present (except the focal animal) were the bystanders. Only one potential partner was chosen in each session, as we focus only on the first choice of the focal.

We augmented our dataset to determine dominance ranks and social relationships, using focal observations of grooming, aggressions, proximity, pant grunts and supplants collected by trained observers (A.M., J.F.G., A.P., L.S.) and field assistants for the Taï Chimpanzee Project's long-term database. Dominance ranks of all communities were calculated using a modification of the Elo rating method (see [43] for details). We used unidirectional pant grunt vocalizations in chimpanzees and non-aggressive supplants in sooty mangabeys to establish hierarchies [43]. Ordinal ranks were standardized daily between 0 and 1. We used two different rank variables per individual: one is the *global rank*, which is independent of party composition and describes an individual's dominance rank in comparison to the whole community. We also considered the *relative rank*, which is the rank they had in comparison to all individuals present at the time of the grooming decision. We calculated dyadic affiliation strength using the Dynamic Dyadic Sociality Index (DDSI, [43,63]) with data collected in the three communities between January 2012 (January 2014 for the mangabeys) and May 2015 (see [43] for details). Much like the Elo rating for rank, the DDSI represents relationships between two individuals dynamically. The dyadic value increases after socio-positive interactions and decreases after socio-negative interaction, allowing us to calculate a daily relationship value for each dyad based on past interactions. We used the duration of grooming exchanges, and resting and feeding in close proximity (1 m or less), as socio-positive, and directed aggression as socio-negative behaviours [64]. The DDSI value of any dyad was extracted for the day before grooming interactions, to make the relationship value independent from the bout in question. Similar to the rank variables, dyads had global and relative social relationship strength. We selected the highest relationship value each potential partner had with any bystander (i.e. any individual who was present but not the focal or that potential partner) as the ‘maximum DDSI’ to control for the presence of close friends.

### 2.2. Models and statistical analysis

We fitted multiple Generalized Linear Mixed Models (GLMM [65]) with binomial error structure and logit link function [66], implemented with R statistical software [67] using the package lme4 [68] to test the impact of different dyadic and bystander variables on grooming partner choice (see electronic supplementary material, table S1 for model parameters). The binomial dependent variable coded whether a potential partner was selected or not. For all models, we included the focal individual's rank in interaction with the squared partner rank. The squared partner rank was chosen as it allows the representation of both grooming of closely ranked partners (which would follow an inverted U-shaped distribution around the focal rank) and high-ranking partners. We included the DDSI of the dyad to test whether they targeted individuals with whom they had previously groomed or had a strong social relationship. We included the reproductive state of the potential partner (female with infant below 3 months of age, maximally tumescent female, other) as a predictor to test whether there was attraction to females with young infants [21–23] or trade of grooming for mating opportunities [14]. As grooming is used to reconcile following aggression in both species [69,70], we included a variable testing whether there was an aggression between the focal individual and potential partner in the 30 min before the grooming session started. Comprehensive kinship data for adults were only available for the South community; in East, no adult maternal kin were present and for the mangabeys, only mother–daughter dyads were known from microsatellite analyses (A Mielke & J Lester 2016, unpublished data), potentially biasing the results. Thus, we could not test the impact of kinship on partner choice. Male mangabeys do not groom each other and did not initiate grooming with females in our dataset, while both male and female chimpanzees did. This limited our ability to control for the sex of the focal individual when comparing the two species. We thus ran two sets of models. In Model 1, we compared the three communities, controlling for the interaction between focal individual's sex and partner sex, and the interaction between partner sex and group identity; in this model, all predictors described above were entered in interaction with group identity. In Model 2, we tested for different patterns in the two chimpanzee sexes, by removing the mangabeys and including the three-way interaction between the sexes of focal individuals and potential partner with group identity. All other predictors were entered in interaction with the focal individual's sex. If the effects of predictors showed group differences between South and East community in Model 1, the interaction with group was included in Model 2. The exception here was grooming a previous aggression opponent, as not enough cases existed in female chimpanzees.

The impact of partner rank can occur on two levels: Individuals have a global rank that describes their status in the community, and a relative rank in the party, capturing shifting partner value based on availability. The global and relative ranks are highly correlated and can therefore not be included in the same model; however, they might still vary in meaningful ways. For example, only 6 out of 52 potential partners in our sample were ever the highest-ranking individual by global rank, but 27 individuals at some point held the highest relative rank in the party. The same was true for the global and relative relationship strength. We fitted both Model 1 and Model 2 twice, once with the global ranks and DDSI of focal individual and partner, and once with their relative ranks and DDSI. We can use differences between global and relative rank/DDSI models to understand whether focal individuals represent potential partners independent of the social environment (if global and relative variables show the same effect), or whether they represent individuals flexibly based on the availability of other options (if the effects of relative and global variables show different patterns). We thus created two models to test the effect of multiple dimensions on grooming partner choice, and fitted each of them twice, using the global and relative variables, respectively. Model 1 consisted of each combination of grooming bouts with all potential partners for *n* = 1529 grooming initiations over *n* = 8809 potential partners. We tested whether those individuals who were chosen differed significantly from those who were not, and if there were group differences. Model 2, focusing only on the chimpanzees, consisted of *n* = 1372 grooming initiations and *n* = 7781 potential partners. We tested whether chimpanzee males and females followed the same patterns when choosing grooming partners.

We included the identities of the focal individual (*N* = 32) and potential partners (*N* = 52), and their dyad combinations (*N* = 342) in models as random effects [71]. Additionally, we included an index for each grooming session as a random effect to account for the non-independence of partner choice within each bout. We initially included all possible random slopes of quantitative fixed within random effects to keep type I error rate at the nominal level of 5% [72]. To reduce model complexity, we identified random slopes that did not show any variance in the full model and removed these [73], using likelihood ratio tests to ascertain that removing random slopes did not significantly change the full model. In all models, we included an offset term for the (log-transformed) inverted number of potential partners to control for differences in choice likelihood in parties of different sizes. Quantitative predictors were z-standardized to a mean of zero and a standard deviation of one [74]. For each model, we conducted a full null model comparison [72] using a likelihood ratio test [75], where the null model included only the control predictors (focal and partner sex, group identity), to test whether the test variables collectively had a significant effect. The null model had the same random effect structure as the full model and it also included the offset term. We tested the significance of the interactions, lower-order interactions, and main effects by systematically dropping them from the model one at a time [75] and comparing the resulting model with the full model using the ‘drop1’ function in R [67]. Variance Inflation Factors (VIF) [76] were derived using the function vif of the R-package ‘car’ [77] applied to a standard linear model excluding the random effects and the interactions for each of the models. Collinearity only existed between the global rank variables and individual sexes, as males are generally higher-ranking than females in both species (maximum VIF = 4.3) [76]. We checked for issues of model stability by excluding levels of all random effects one at a time from the data and fitting the same models to these subsets. This indicated that the relative paucity of cases for previous aggressions in mangabeys (*n* = 20 cases) and females with young offspring in East (*n* = 36 cases) led to relative uncertainty in the estimates for these variables in interaction with group identity.

## 3. Results

In the mangabeys, adult males did not initiate any grooming interactions, neither with each other nor with adult females. Consequently, when reporting results below, ‘mangabeys’ only concerns female individuals. Female mangabeys initiated grooming at the same rate as male chimpanzees (around 0.3 interactions per hour, see table 1). While chimpanzee females initiated less grooming than males, they still did so at relatively high rates (0.1–0.2 interactions per hour). However, there were not sufficient grooming initiations of female chimpanzees in the aftermath of aggressions to test whether they preferentially chose previous aggression partners. Decisions were made in highly variable social environments: in the mangabeys, 157 grooming decisions took place in 156 unique sets of bystanders; in East chimpanzees, 640 decisions with 438 unique sets of bystanders; in South chimpanzees, 732 decisions with 451 unique sets of bystanders.

### 3.1. Model 1—all groups

**Figure 1.**
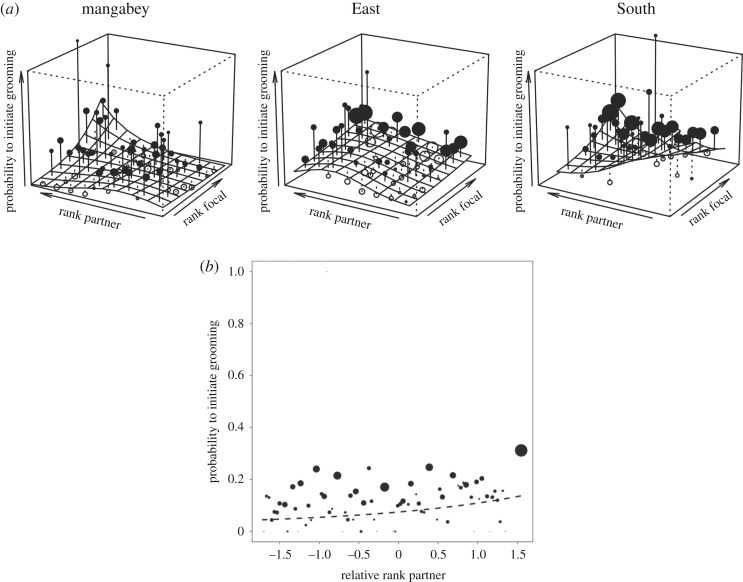
(*a*) Likelihood that the focal individual initiates grooming with a potential partner depending on global focal and partner dominance ranks in sooty mangabeys (left), East (centre) and South (right) chimpanzee communities (Model 1—Global Ranks). Higher rank values indicate increasing rank. Shown is the model result (surface) for a party of average size (larger point volumes denote a larger number of observations [range 1–520 observations]; black points indicate residuals larger than average). Mangabeys and South community show an attraction to closely ranked partners, low-ranking individuals in the East community to medium-ranked, high-ranking to high-ranking individuals. (*b*) Likelihood that the focal individual initiates grooming with a potential partner depending on the relative partner rank (Model 1—Relative Ranks). Higher rank values indicate increased rank. Shown are the observed probabilities to initiate grooming in a party of average size (larger point areas denote a larger number of observations [range 1–1187 observations]) as well as the model result (line). Higher relative partner rank increased the likelihood of grooming. No group differences were found.

The full null model comparisons of both models using global and relative rank for all three communities were significant (Likelihood ratio tests: Global Model: *X*^2^ = 144.93, d.f. = 30, *p* < 0.001, electronic supplementary material, table S2A; Relative Model: *X*^2^ = 22.71, d.f. = 30, *p* < 0.001, electronic supplementary material, table S3A). In the Global Model, the interaction between the squared global partner rank with global focal rank (*X*^2^ = 5.14, d.f. = 1, *p* = 0.023; electronic supplementary material, table S2B) and the three-way interaction between global partner rank, global focal rank and group (*X*^2^ = 8.63, d.f. = 2, *p* = 0.014) were significant. Taken together, these interactions revealed that in the mangabeys, there was an attraction to closely ranked partners, especially in high-ranking individuals (figure 1*a*). In the East community, low-ranking initiators targeted medium-ranked partners, while medium- and high-ranking initiators focused on closely ranked partners. In the South community, individuals targeted closely ranked partners, and this effect was stronger in low-ranking individuals. In the Relative Model, no interactions of rank were significant and the main effect of relative partner rank revealed that focal individuals in all three communities targeted partners who were high-ranking within the party (*X*^2^ = 10.91, d.f. = 1, *p* = 0.001; figure 1*b*; electronic supplementary material, table S3B).

**Figure 2.**
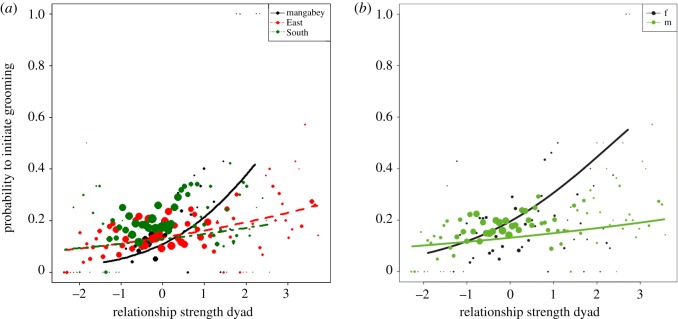
Likelihood that focal individuals initiate grooming with a potential partner depending on the value of their social relationship, in interaction with group identity (*a*, Model 1—Global Ranks) and in interaction with focal sex within the chimpanzees (*b*, Model 2—Global Ranks). Shown are the observed probabilities to initiate grooming in a party of average size (larger point areas denote a larger number of observations [range 1–181 observations]) as well as the model result (lines). Mangabey and chimpanzee females strongly preferred potential partners with whom they had a high social relationship value.

Both models showed a significant interaction between global/relative relationship value and group, respectively (Global Model: *X*^2^ = 5.93, d.f. = 2, *p* = 0.052; Relative Model: *X*^2^ = 6.64, d.f. = 2, *p* = 0.036). Focal individuals in all groups targeted close social partners for grooming, but this effect was more pronounced in the mangabeys (figure 2*a*). There was also a significant main effect for the reproductive state of the partner in the Global Model (*X*^2^ = 39.52, d.f. = 2, *p* < 0.001) and the Relative Model (*X*^2^ = 39.75, d.f. = 2, *p* < 0.001). In both chimpanzee communities and the mangabeys, focal individuals preferably initiated grooming with females with infants less than 3 months of age (electronic supplementary material, figure S1).

**Figure 3.**
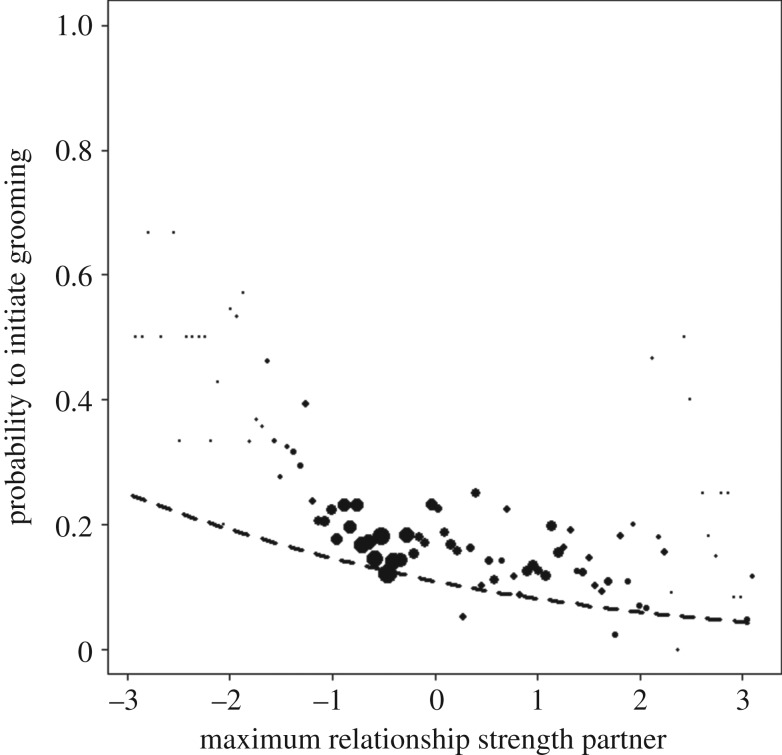
Likelihood that the focal individual initiates grooming with a potential partner depending on the maximum relationship strength of the partner with any bystander (Model 1—Global Ranks). Shown are the observed probabilities to initiate grooming in a party of average size (larger point areas denote a larger number of observations [range 1–472 observations]) as well as the model result (line). Individuals preferred potential partners who had low maximum relationship strength with bystanders. No group differences were found.

There were no group differences related to the effects of the bystander relationships in either model. The maximum DDSI of partner (Global Model: *X*^2^ = 19.13, d.f. = 1, *p* < 0.001; Relative Model: *X*^2^ = 10.63, d.f. = 1, *p* = 0.001; figure 3) was significant in both models and revealed that individuals in all three communities preferably initiated grooming with individuals who did not have any strong social partners present. In both models, the main effect of recent aggression between focal individuals and partner was significant (Global Model: *X*^2^ = 10.33, d.f. = 1, *p* = 0.006; Relative Model: *X*^2^ = 16.84, d.f. = 1, *p* < 0.001; electronic supplementary material, figure S2). Focal individuals preferably targeted individuals with whom they had an aggression in the 30 min prior.

### 3.2. Model 2—chimpanzees

The full null model comparisons of both the models using global and relative rank for the two chimpanzee communities were significant (Global Model: *X*^2^ = 110.08, d.f. = 30, *p* < 0.001, electronic supplementary material, table S4A; Relative Model: *X*^2^ = 101.77, d.f. = 20, *p* < 0.001, electronic supplementary material, table S5A). The results for global ranks matched those of Model 1 (electronic supplementary material, table S4B). For the relative rank, male chimpanzees continued to show attraction to individuals with high relative rank, while females showed a bias towards individuals who were close to them in relative rank (Relative Model: *X*^2^ = 3.45, d.f. = 1, *p* = 0.063; electronic supplementary material, table S5B and figure S3). Both models showed a significant interaction between the relationship value of the dyad and focal sex (Global Model: *X*^2^ = 6.68, d.f. = 1, *p* = 0.010; Relative Model: *X*^2^ = 5.47, d.f. = 1, *p* = 0.019), with female chimpanzees targeting partners with whom they had a closer social relationship, while males did not or only weakly show this effect (figure 2*b*). The maximum DDSI of the partner in both models showed no sex differences and results matched those of Model 1. The effect of previous aggressions also matched the results of Model 1. In the Global Model, we found a significant interaction between the sex of the focal and the sex of the partner (*X*^2^ = 15.42, d.f. = 1, *p* < 0.001), with females in both communities preferably initiating grooming with males, but no specific preferences in males. In the Relative Model, there was a significant interaction between the two sex variables and group identity (*X*^2^ = 7.71, d.f. = 1, *p* = 0.006), indicating that while the aforementioned effect persisted in the East community, there were no preferences of focal individuals for either sex in South.

## 4. Discussion

In this study, we conceptualized grooming as a decision-making event where individual chimpanzees and sooty mangabeys selected a partner from a group of available individuals. This allowed us to test the flexibility of grooming partner choice with regard to the number of variables both species integrate. We found that multiple partner attributes had an impact on partner selection, but also that the availability of alternative partners and their relationships influenced partner choice. Individuals avoided grooming partners that had strong social relationships with at least one bystander, suggesting these primates use flexible decision-making that assesses a partner's value given the local social environment. Partners are associated with different benefits and risks, and it seems that individuals in both species flexibly distributed their grooming to maximize benefits and minimize costs.

Our results suggest that focal individuals in both species incorporated a number of different variables into their grooming decisions. Females in both species preferably initiated grooming with potential partners with whom they already had a strong social relationship, either due to a preference for bond partners [78] or as reciprocation for previous grooming [52,79,80]. Male chimpanzees were less discerning in their partner choice with regard to their existing relationship with other group members [58], which corresponds to association patterns in these communities [81]. As our results reflect grooming initiations, but ignore subsequent grooming duration, it is likely that male chimpanzees initiate grooming with a large number of partners for various reasons, but focus long grooming bouts on particular bond partners [82]. Male mangabeys did not groom each other at all, and did not initiate grooming with females even though they reciprocate it within bouts, indicating that there might be limited value of exchanging grooming for other commodities. Mangabeys exhibit strong female reproductive synchrony and females are highly promiscuous [83]. Grooming might thus have limited value for coalition formation among mangabey males for either consortship takeover [84] or concessions for mating access [85]. Individuals preferred grooming previous aggression opponents, confirming that both species use grooming for reconciliation [69,70]; however, for the mangabeys this result was based on a small sample size and is thus quite uncertain, and should be verified using more traditional tests of reconciliation [69]. While a grooming bias towards females with young infants has been reported in cercopithecine species [86], our results indicate that both male and female chimpanzees might be attracted to mothers with newborn infants too. We were only able to assess a limited number of partner attributes, suggesting both chimpanzee and mangabeys are assessing, remembering and integrating even more variables into their grooming decisions.

In both species, the wider social environment also influenced partner choice. Groomers in mangabeys and South chimpanzees focused their efforts on individuals that were close in global rank, as did medium- and high-ranking individuals in East chimpanzees, while low-ranking individuals in East chose medium-ranked partners. However, locally, focal individuals in both species preferably initiated grooming with individuals that had a high relative rank compared to other potential partners. Only female chimpanzees also preferred partners with a similar relative rank. This divergence between the two rank variables indicates that either, initiators were attracted to the highest-ranking individual available [51], but association is biased towards closely ranked group members; or, in line with Seyfarth's priority of access model [50], there is competition for high-ranking grooming partners, and low-ranking individuals use the absence of higher-ranking group members to gain access to preferred partners [12,44]. Alternatively, the absence of high-ranking bystanders could reduce the likelihood that the partner defects in the chimpanzees [44] or interventions occur in the mangabeys [43]. Our results indicate that targets did not have one fixed value as grooming partners based on their rank, but rather that their value depended on local conditions and was judged flexibly by other group members. This could allow individuals, especially in communities exhibiting fission–fusion dynamics, to fine-tune their behavioural strategies to their own local market value [9]. These results also have implications for the interpretation of studies using aggregated across-dyad correlations to test grooming partner choice, highlighting the importance of accounting for association patterns [87]. While a bias to groom closely ranked individuals that has often been observed in studies aggregating grooming in primate communities [50,88], this might be at least partially an emergent property of increased spatial proximity to closely ranked individuals [89].

The presence of close social partners of a grooming target might make it more likely that these individuals intervene into the grooming bout [43] or that the groomee switches partners [44], causing the groomer to lose their initial investment. Grooming initiators in both species preferred individuals who had no close social partners among the bystanders. Alternatively, individuals may actively seek out partners for whom grooming is currently unlikely, as the relatively low local supply of grooming partners might make the service more valuable for the receiver [7]. Grooming interventions are common in both species [43], making it unlikely that this result is driven purely by potential partners with strong relationship values already being engaged in grooming. Importantly, the avoidance of grooming partners who have strong social partners present indicates that both species are able to inhibit their own attraction to individuals based on the social environment, suggesting that they possess the cognitive abilities to suppress ineffective responses [13,38,45]. These results also add to the growing literature on triadic awareness of social relationships in non-human animals [43,90–94], and suggest that individuals are able to keep track of multiple dyadic and triadic relationships simultaneously, and determine the relative value of each. Future research should investigate whether the choice of when and whom to groom is being used as image scoring [95], for reputation management [96,97], or to elicit indirect reciprocity [98,99].

Though we predicted species differences in the bystander variables and the impact of rank on partner choice, due to the differences in hierarchy steepness [55,100] and fission–fusion patterns, our results suggest that even mangabeys, which exhibit relatively low levels of fission–fusion dynamics, made flexible, situation-specific grooming decisions. This indicates that cognitive skills supposedly necessary for fission–fusion systems [13,45] might be more widespread than previously appreciated. Individuals chose grooming partners based on their own needs, but do so within the constraints placed by a local social environment [3]. While we focus on the decision whom to groom, future research should integrate this with the questions of when to groom in the first place and the reaction of the receiver based on the social environment, to further elucidate how strongly bystanders influence behavioural decision-making. Integrating more situational variables into the analysis, e.g. the possession of resources at the time of grooming, could give further insight into the role of demand and supply of commodities in grooming decisions.

When discussing the cognitive requirements facing cooperating animals, researchers have focused on the challenge of representing previous interactions of the cooperators [17,101]. Here, we show the high behavioural flexibility and possibly cognition needed to optimize partner choice [13,102]. Flexibly adjusting partner choice allows individuals to optimize their own benefits over multiple time scales, sometimes choosing partners because of the immediate benefit they offer (e.g. to repair relationships after aggression or to get access to an infant), sometimes reciprocating previous grooming, and sometimes for future benefits (such as coalitionary support). While the cognitive requirements of keeping track of social relationships are still debatable [17,102], viewing partner choice in grooming as a value-based decision-making process, partially based on the knowledge of third-party relationships, allows us to compare how flexibly different species solve similar social problems.

## Supplementary Material

Mielke et al. ESM Grooming Decisions

## Data accessibility

Grooming decision data for all models, and model scripts are available at the Dryad Digital Repository: http://dx.doi.org/10.5061/dryad.t8c88vh [103].
