# Supplementary material for: Flexible decision-making in grooming partner choice in sooty mangabeys and chimpanzees
Source: R Soc Open Sci. 2018 Jul 11;5(7):172143. doi: 10.1098/rsos.172143 (PMC6083658; doi:10.1098/rsos.172143)
Supplement: Mielke et al. ESM Grooming Decisions [file rsos172143supp1.doc]

**Proceedings of the Royal Society**

**Flexible decision making in grooming partner choice in sooty mangabeys and chimpanzees**

**Alexander Mielke^1,2^, Anna Preis^1,2^, Liran Samuni^1,2^, Jan F. Gogarten^2,3,4^, Roman M. Wittig^1,2,a^, Catherine Crockford ^1,2,a^**

1 Max Planck Institute for Evolutionary Anthropology, Department of Primatology, Leipzig, Germany

2 Taï Chimpanzee Project, Centre Suisse de Recherches Scientifiques en Côte d'Ivoire, Abidjan, Côte d'Ivoire

3 McGill University, Department of Biology, Montreal, Canada

4 Robert Koch Institute, P3: “Epidemiology of Highly Pathogenic Microorganisms”, Berlin, Germany

a C.C. and R.M.W. contributed equally to this work.

**Electronic Supplementary Material**

**Table S1: Overview of model parameters present in the models fitted (Global = in comparison to whole community, Relative = in comparison to party, DDSI = Dynamic Dyadic Sociality Index)**

| **Model** | **Test Parameters** | **Control Parameters** | **Random Effects** |
| --- | --- | --- | --- |
| 1 – Global | Global Rank Focal * Global Rank Partner ^2^ * Group +  Global DDSI Dyad * Group +  Reproductive State Partner * Group + Previous Aggression Dyad * Group +  Maximum DDSI Partner * Group | Sex Partner * Group +  Sex Focal * Sex Partner + Offset (log(Party Size-1)) | ID Focal  ID Potential Partner  ID Dyad  ID Grooming Bout  Random Slopes |
| 1 – Relative | Relative Rank Focal * Relative Rank Partner ^2^ * Group +  Relative DDSI Dyad * Group +  Reproductive State Partner * Group + Previous Aggression Dyad * Group +  Maximum DDSI Partner * Group | Sex Partner * Group +  Sex Focal * Sex Partner + Offset (log(Party Size-1)) | ID Focal  ID Potential Partner  ID Dyad  ID Grooming Bout  Random Slopes |
| 2 – Global | Global Rank Focal * Global Rank Partner ^2^ * Group * Sex Focal +  Global DDSI Dyad * Sex Focal +  Reproductive State Partner * Sex Focal + Previous Aggression Dyad * Group +  Maximum DDSI Partner * Sex Focal | Sex Focal * Sex Partner* Group +  Offset (log(Party Size-1)) | ID Focal  ID Potential Partner  ID Dyad  ID Grooming Bout  Random Slopes |
| 2 – Relative | Relative Rank Focal * Relative Rank Partner ^2^ * Sex Focal +  Relative DDSI Dyad * Sex Focal +  Reproductive State Partner * Sex Focal + Previous Aggression Dyad * Group +  Maximum DDSI Partner * Sex Focal | Sex Focal * Sex Partner* Group +  Offset (log(Party Size-1)) | ID Focal  ID Potential Partner  ID Dyad  ID Grooming Bout  Random Slopes |


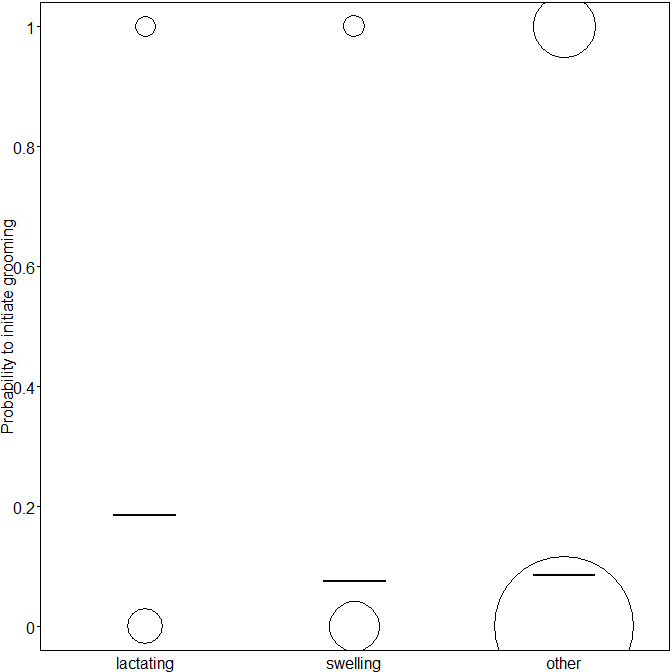


*Figure S1: Likelihood of the focal individual to initiate grooming with a potential partner and its dependency on the reproductive state of the partner (Model 1 – Global Ranks). Partners are either females with infants below 3 months of age (“lactating”), females with sexual swellings (“swelling”), or males and other females. Shown are the observed probabilities to initiate grooming in a party of average size (larger bubble areas denote a larger number of observations, range 141 to 6073 observations) as well as the model result (lines). Individuals were more likely to initiate grooming with mothers of small infants.*


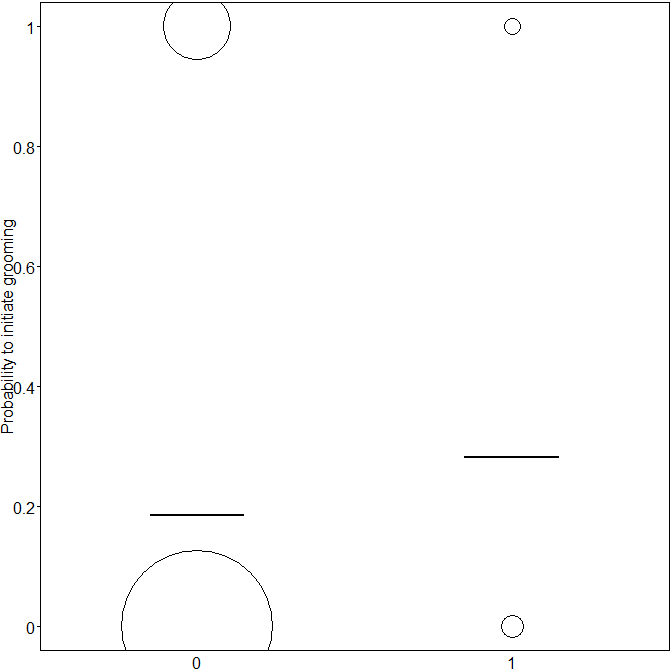


*Figure S2: Likelihood of the focal individual to initiate grooming with a potential partner and its dependency on whether they had an aggressive interaction in the past 30min (“1”) or not (“0”) (Model 1 – Global Ranks). Shown are the observed probabilities to initiate grooming in a party of average size (larger bubble areas denote a larger number of observations, range 86 to 7118 observations) as well as the model result (lines). Individuals were more likely to initiate grooming after aggressions.*


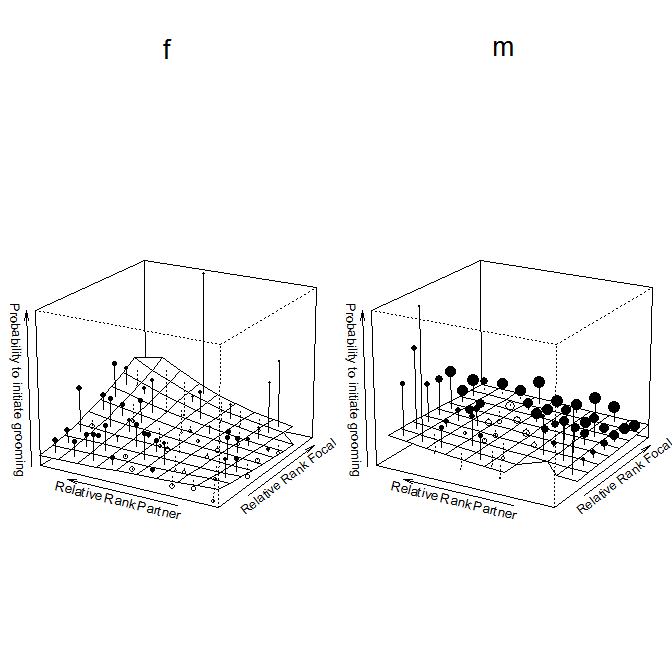


*Figure S3: Likelihood of the focal individual in chimpanzees to initiate grooming with a potential partner and its dependency on the interaction of relative focal rank and relative partner rank in female (left) and male (right) chimpanzees (Model 2 – Relative Ranks). Higher rank values indicate increasing rank. Shown is the model result (surface) for a party of average size (larger point volumes denote a larger number of observations [range 1 to 296 observations]). Females were more likely to initiate grooming with individuals who were close to them in relative rank, while males chose partners who were high in relative rank in the party.*

**Table S2A: Result of Full Model 1 - Global: Comparing grooming partner choice in mangabeys and chimpanzees, using community-wide rank and relationship variables. Test parameters in bold. Estimate and Standard Error from full model, X², df and p-values result from a likelihood ratio test comparing the reduced model lacking this term with the full model. DDSI = Dynamic Dyadic Sociality Index. Full null model comparison: X² = 154.94, df = 33, p < 0.001**

| **Term** | **Estimate** | **SE** | **Χ^2^** | **df** | **p** |
| --- | --- | --- | --- | --- | --- |
| Intercept | -0.370 | 0.385 | (1) | (1) | (1) |
| Group East ^(3)^ | 0.124 | 0.497 | (1) | (1) | (1) |
| Group South ^(3)^ | -0.288 | 0.496 | (1) | (1) | (1) |
| Reproductive State Partner Lactating ^(4)^ | 0.832 | 0.235 | (1) | (1) | (1) |
| Reproductive State Partner Swelling ^(4)^ | 0.671 | 0.433 | (1) | (1) | (1) |
| Sex Partner (female=0, male=1) | 0.537 | 0.717 | (1) | (1) | (1) |
| Sex Focal (female=0, male=1) | 0.702 | 0.288 | (1) | (1) | (1) |
| Global Rank Focal ^(5)^ | 0.157 | 0.166 | (1) | (1) | (1) |
| Global Rank Partner ^(5)^ | 0.659 | 0.340 | (1) | (1) | (1) |
| Global Rank Partner ^2^ | 0.075 | 0.260 | (1) | (1) | (1) |
| Global DDSI Dyad ^(5)^ | 0.760 | 0.201 | (1) | (1) | (1) |
| Maximum DDSI Partner ^(5)^ | -0.308 | 0.181 | (1) | (1) | (1) |
| Previous Aggression (no=0, yes=1) | -0.359 | 0.652 | (1) | (1) | (1) |
| Global Rank Focal * Global Rank Partner | 0.429 | 0.147 | (1) | (1) | (1) |
| Global Rank Focal * Global Rank Partner ^2^ | 0.089 | 0.119 | (1) | (1) | (1) |
| Global Rank Focal * Group East | -0.228 | 0.223 | (1) | (1) | (1) |
| Global Rank Focal * Group South | -0.371 | 0.240 | (1) | (1) | (1) |
| Global Rank Partner * Group East | -0.484 | 0.425 | (1) | (1) | (1) |
| Global Rank Partner * Group South | -0.592 | 0.417 | (1) | (1) | (1) |
| Global Rank Partner^2^ * Group East | -0.291 | 0.302 | (1) | (1) | (1) |
| Global Rank Partner^2^ * Group South | 0.175 | 0.293 | (1) | (1) | (1) |
| **Global DDSI Dyad * Group East** | -0.542 | 0.216 | 8.04 | 2 | 0.018 |
| **Global DDSI Dyad * Group South** | -0.582 | 0.213 |  |  |  |
| **Reproductive State Partner Lactating * Group East** | 0.967 | 0.575 | 8.79 | 4 | 0.067 |
| **Reproductive State Partner Lactating * Group South** | 0.103 | 0.316 |  |  |  |
| **Reproductive State Partner Swelling * Group East** | -1.031 | 0.471 |  |  |  |
| **Reproductive State Partner Swelling * Group South** | -0.701 | 0.466 |  |  |  |
| **Maximum DDSI Partner * Group East** | 0.121 | 0.192 | 1.39 | 2 | 0.500 |
| **Maximum DDSI Partner * Group South** | 0.192 | 0.192 |  |  |  |
| **Previous Aggression * Group East** | 0.638 | 0.706 | 5.80 | 2 | 0.055 |
| **Previous Aggression * Group South** | 1.152 | 0.682 |  |  |  |
| Sex Partner * Group East | 0.719 | 0.903 | 0.58 | 2 | 0.748 |
| Sex Partner * Group South | 0.716 | 0.900 |  |  |  |
| Sex Partner * Sex Focal | -1.780 | 0.407 | 18.82 | 1 | 0.000 |
| Global Rank Focal * Global Rank Partner * Group East | -0.403 | 0.192 | (1) | (1) | (1) |
| Global Rank Focal * Global Rank Partner * Group South | 0.138 | 0.208 | (1) | (1) | (1) |
| **Global Rank Focal * Global Rank Partner^2^ * Group East** | 0.081 | 0.155 | 0.42 | 2 | 0.809 |
| **Global Rank Focal * Global Rank Partner^2^ * Group South** | 0.101 | 0.171 |  |  |  |

1. = not shown because of having a very limited interpretation
2. = control predictor
3. = reference level is Mangabey
4. = reference level is Other
5. = z-transformed, mean and standard deviation in Table S6

**Table S2B: Result of Final Model 1 - Global: Comparing grooming partner choice between mangabeys and chimpanzees, using community-wide rank and relationship variables. Test parameters in bold. Estimate and Standard Error from full model, X², df and p-values from a likelihood ratio test comparing the reduced model lacking this term with the full model. DDSI = Dynamic Dyadic Sociality Index.**

| **Term** | **Estimate** | **SE** | **Χ^2^** | **df** | **p** |
| --- | --- | --- | --- | --- | --- |
| Intercept | -0.303 | 0.281 | (1) | (1) | (1) |
| Group East ^(3)^ | -0.103 | 0.412 | (1) | (1) | (1) |
| Group South ^(3)^ | -0.279 | 0.409 | (1) | (1) | (1) |
| **Reproductive State Partner Lactating ^(4)^** | 0.900 | 0.226 | 39.52 | 2 | 0.000 |
| **Reproductive State Partner Swelling ^(4)^** | -0.129 | 0.122 |  |  |  |
| Sex Partner (female=0, male=1) | 0.560 | 0.618 | (1) | (1) | (1) |
| Sex Focal (female=0, male=1) | 0.713 | 0.288 | (1) | (1) | (1) |
| Global Rank Focal ^(5)^ | 0.139 | 0.139 | (1) | (1) | (1) |
| Global Rank Partner ^(5)^ | 0.716 | 0.294 | (1) | (1) | (1) |
| Global Rank Partner ^2^ | 0.104 | 0.084 | (1) | (1) | (1) |
| Global DDSI Dyad ^(5)^ | 0.710 | 0.199 | (1) | (1) | (1) |
| **Maximum DDSI Partner ^(5)^** | -0.159 | 0.045 | 10.46 | 1 | 0.001 |
| **Previous Aggression (no=0, yes=1)** | **0.543** | **0.155** | 11.84 | 1 | 0.001 |
| Global Rank Focal * Global Rank Partner | 0.483 | 0.141 | (1) | (1) | (1) |
| **Global Rank Focal * Global Rank Partner ^2^** | 0.133 | 0.059 | 5.14 | 1 | 0.002 |
| Global Rank Focal * Group East | -0.200 | 0.181 | (1) | (1) | (1) |
| Global Rank Focal * Group South | -0.310 | 0.194 | (1) | (1) | (1) |
| Global Rank Partner * Group East | -0.426 | 0.373 | (1) | (1) | (1) |
| Global Rank Partner * Group South | -0.643 | 0.376 | (1) | (1) | (1) |
| **Global DDSI Dyad * Group East** | -0.508 | 0.210 | 5.93 | 2 | 0.052 |
| **Global DDSI Dyad * Group South** | -0.531 | 0.211 |  |  |  |
| Sex Partner * Group East | 0.457 | 0.791 | 1.15 | 2 | 0.562 |
| Sex Partner * Group South | 0.744 | 0.793 |  |  |  |
| Sex Partner * Sex Focal | -1.781 | 0.404 | 19.09 | 1 | 0.000 |
| **Global Rank Focal * Global Rank Partner * Group East** | -0.410 | 0.185 | 8.63 | 2 | 0.013 |
| **Global Rank Focal * Global Rank Partner * Group South** | 0.071 | 0.201 |  |  |  |

1. = not shown because of having a very limited interpretation
2. = control predictor
3. = reference level is Mangabey
4. = reference level is Other
5. = z-transformed, mean and standard deviation in Table S6

**Table S3A: Result of Full Model 1 - Relative: Comparing grooming partner choice between mangabeys and chimpanzees, using party-specific rank and relationship variables. Test parameters in bold. Estimate and Standard Error from full model, X², df and p-values from a likelihood ratio test comparing the reduced model lacking this term with the full model. DDSI = Dynamic Dyadic Sociality Index. Full null model comparison: X² = 129.13, df = 33, p < 0.001**

| **Term** | **Estimate** | **SE** | **Χ^2^** | **df** | **p** |
| --- | --- | --- | --- | --- | --- |
| Intercept | -0.629 | 0.296 | (1) | (1) | (1) |
| Group East ^(3)^ | 0.315 | 0.388 | (1) | (1) | (1) |
| Group South ^(3)^ | 0.110 | 0.410 | (1) | (1) | (1) |
| Reproductive State Partner Lactating ^(4)^ | 0.822 | 0.240 | (1) | (1) | (1) |
| Reproductive State Partner Swelling ^(4)^ | 0.677 | 0.456 | (1) | (1) | (1) |
| Sex Partner (female=0, male=1) | 0.293 | 0.536 | (1) | (1) | (1) |
| Sex Focal (female=0, male=1) | 0.327 | 0.214 | (1) | (1) | (1) |
| Relative Rank Focal ^(5)^ | -0.013 | 0.153 | (1) | (1) | (1) |
| Relative Rank Partner ^(5)^ | 0.428 | 0.187 | (1) | (1) | (1) |
| Relative Rank Partner ^2^ | -0.017 | 0.181 | (1) | (1) | (1) |
| Relative DDSI Dyad ^(5)^ | 0.495 | 0.126 | (1) | (1) | (1) |
| Maximum DDSI Partner ^(5)^ | -0.210 | 0.181 | (1) | (1) | (1) |
| Previous Aggression (no=0, yes=1) | 0.008 | 0.671 | (1) | (1) | (1) |
| Relative Rank Focal * Relative Rank Partner | 0.143 | 0.134 | (1) | (1) | (1) |
| Relative Rank Focal * Relative Rank Partner ^2^ | 0.129 | 0.131 | (1) | (1) | (1) |
| Relative Rank Focal * Group East | 0.035 | 0.185 | (1) | (1) | (1) |
| Relative Rank Focal * Group South | 0.215 | 0.185 | (1) | (1) | (1) |
| Relative Rank Partner * Group East | -0.183 | 0.219 | (1) | (1) | (1) |
| Relative Rank Partner * Group South | -0.238 | 0.211 | (1) | (1) | (1) |
| Relative Rank Partner^2^ * Group East | 0.019 | 0.200 | (1) | (1) | (1) |
| Relative Rank Partner^2^ * Group South | 0.117 | 0.195 | (1) | (1) | (1) |
| **Relative DDSI Dyad * Group East** | -0.358 | 0.147 | 5.88 | 2 | 0.053 |
| **Relative DDSI Dyad * Group South** | -0.267 | 0.141 |  |  |  |
| **Reproductive State Partner Lactating * Group East** | 1.138 | 0.585 | 8.64 | 4 | 0.071 |
| **Reproductive State Partner Lactating * Group South** | 0.233 | 0.327 |  |  |  |
| **Reproductive State Partner Swelling * Group East** | -0.983 | 0.495 |  |  |  |
| **Reproductive State Partner Swelling * Group South** | -0.710 | 0.492 |  |  |  |
| **Maximum DDSI Partner * Group East** | 0.029 | 0.191 | 0.53 | 2 | 0.766 |
| **Maximum DDSI Partner * Group South** | 0.078 | 0.191 |  |  |  |
| **Previous Aggression * Group East** | 0.283 | 0.730 | 3.58 | 2 | 0.167 |
| **Previous Aggression * Group South** | 0.865 | 0.703 |  |  |  |
| Sex Partner * Group East | 0.437 | 0.679 | 4.59 | 2 | 0.101 |
| Sex Partner * Group South | 0.497 | 0.685 |  |  |  |
| Sex Partner * Sex Focal | -0.926 | 0.322 | 7.69 | 1 | 0.001 |
| Relative Rank Focal * Relative Rank Partner * Group East | -0.214 | 0.164 | (1) | (1) | (1) |
| Relative Rank Focal * Relative Rank Partner * Group South | -0.072 | 0.162 | (1) | (1) | (1) |
| **Relative Rank Focal * Relative Rank Partner^2^ * Group East** | -0.092 | 0.152 | 3.23 | 2 | 0.199 |
| **Relative Rank Focal * Relative Rank Partner^2^ * Group South** | -0.231 | 0.153 |  |  |  |

1. = not shown because of having a very limited interpretation
2. = control predictor
3. = reference level is Mangabey
4. = reference level is Other
5. = z-transformed, mean and standard deviation in Table S6

**Table S3B: Result of Final Model 1 - Relative: Comparing grooming partner choice between mangabeys and chimpanzees, using party-specific rank and relationship variables. Test parameters in bold. Estimate and Standard Error from full model, X², df and p-values from a likelihood ratio test comparing the reduced model lacking this term with the full model. DDSI = Dynamic Dyadic Sociality Index.**

| **Term** | **Estimate** | **SE** | **Χ^2^** | **df** | **p** |
| --- | --- | --- | --- | --- | --- |
| Intercept | -0.653 | 0.187 | (1) | (1) | (1) |
| Group East ^(3)^ | 0.352 | 0.290 | (1) | (1) | (1) |
| Group South ^(3)^ | 0.310 | 0.306 | (1) | (1) | (1) |
| **Reproductive State Partner Lactating ^(4)^** | 0.953 | 0.153 | 39.75 | 2 | 0.000 |
| **Reproductive State Partner Swelling ^(4)^** | -0.127 | 0.129 |  |  |  |
| Sex Partner (female=0, male=1) | 0.335 | 0.469 | (1) | (1) | (1) |
| Sex Focal (female=0, male=1) | 0.278 | 0.206 | (1) | (1) | (1) |
| Relative Rank Focal ^(5)^ | 0.097 | 0.058 | 1.55 | 1 | 0.212 |
| **Relative Rank Partner ^(5)^** | 0.241 | 0.070 | 10.91 | 1 | 0.001 |
| **Relative DDSI Dyad ^(5)^** | 0.495 | 0.125 | (1) | (1) | (1) |
| **Maximum DDSI Partner ^(5)^** | -0.161 | 0.044 | 10.63 | 1 | 0.001 |
| **Previous Aggression (no=0, yes=1**) | 0.625 | 0.163 | 16.84 | 1 | 0.000 |
| **Relative DDSI Dyad * Group East** | -0.357 | 0.147 | 6.64 | 2 | 0.036 |
| **Relative DDSI Dyad * Group South** | -0.268 | 0.141 |  |  |  |
| Sex Partner * Group East | 0.386 | 0.597 | 0.51 | 2 | 0.77 |
| Sex Partner * Group South | 0.397 | 0.599 |  |  |  |
| Sex Partner * Sex Focal | -0.913 | 0.310 | 8.23 | 1 | 0.004 |

1. = not shown because of having a very limited interpretation
2. = control predictor
3. = reference level is Mangabey
4. = reference level is Other
5. = z-transformed, mean and standard deviation in Table S6

**Table S4A: Result of Full Model 2 - Global: Comparing grooming partner choice between chimpanzee males and females, using community-wide rank and relationship variables. Test parameters in bold. Estimate and Standard Error from full model, X², df and p-values from a likelihood ratio test comparing the reduced model lacking this term with the full model. DDSI = Dynamic Dyadic Sociality Index. Full null model comparison: X² = 121.87, df = 32, p < 0.001**

| **Term** | **Estimate** | **SE** | **Χ^2^** | **df** | **p** |
| --- | --- | --- | --- | --- | --- |
| Intercept | -0.179 | 0.752 | (1) | (1) | (1) |
| Group ^(2)^ | 0.252 | 1.053 | (1) | (1) | (1) |
| Reproductive State Partner Lactating ^(3)^ | 0.952 | 0.676 | (1) | (1) | (1) |
| Reproductive State Partner Swelling ^(3)^ | -1.267 | 0.583 | (1) | (1) | (1) |
| Sex Partner (female=0, male=1) | 2.416 | 0.770 | (1) | (1) | (1) |
| Sex Focal (female=0, male=1) | 0.607 | 0.743 | (1) | (1) | (1) |
| Global Rank Focal ^(4)^ | 0.122 | 0.278 | (1) | (1) | (1) |
| Global Rank Partner ^(4)^ | -0.033 | 0.674 | (1) | (1) | (1) |
| Global Rank Partner ^2^ | -0.102 | 0.591 | (1) | (1) | (1) |
| Global DDSI Dyad ^(4)^ | 0.592 | 0.148 | (1) | (1) | (1) |
| Maximum DDSI Partner ^(4)^ | -0.328 | 0.112 | (1) | (1) | (1) |
| Previous Aggression (no=0, yes=1) | 0.286 | 0.270 | (1) | (1) | (1) |
| Global Rank Focal * Global Rank Partner | 0.195 | 0.246 | (1) | (1) | (1) |
| Global Rank Focal * Global Rank Partner ^2^ | 0.215 | 0.246 | (1) | (1) | (1) |
| Global Rank Focal * Group | 0.031 | 0.422 | (1) | (1) | (1) |
| Global Rank Focal * Sex Focal | -0.280 | 0.353 | (1) | (1) | (1) |
| Global Rank Partner * Group | 1.817 | 1.014 | (1) | (1) | (1) |
| Global Rank Partner^2^ * Group | -0.302 | 0.856 | (1) | (1) | (1) |
| Global Rank Partner * Sex Focal | 0.268 | 0.670 | (1) | (1) | (1) |
| Global Rank Partner^2^ * Sex Focal | -0.041 | 0.583 | (1) | (1) | (1) |
| **Global DDSI Dyad * Sex Focal** | -0.444 | 0.157 | 8.01 | 1 | 0.005 |
| **Reproductive State Partner Lactating * Sex Focal** | 0.071 | 0.691 | 5.11 | 2 | 0.077 |
| **Reproductive State Partner Swelling * Sex Focal** | 1.135 | 0.595 |  |  |  |
| **Maximum DDSI Partner * Sex Focal** | 0.201 | 0.115 | 3.12 | 1 | 0.077 |
| **Previous Aggression * Group** | 0.511 | 0.336 | 2.46 | 1 | 0.117 |
| Sex Focal * Group | -0.796 | 1.041 | (1) | (1) | (1) |
| Sex Partner * Group | -2.364 | 1.233 | (1) | (1) | (1) |
| Sex Partner * Sex Focal | -2.943 | 0.767 | (1) | (1) | (1) |
| **Sex Partner * Sex Focal * Group** | 2.560 | 1.246 | 4.29 | 1 | 0.038 |
| Global Rank Focal * Global Rank Partner * Sex Focal | -0.324 | 0.293 | (1) | (1) | (1) |
| Global Rank Focal * Global Rank Partner ^2^ * Sex Focal | -0.104 | 0.288 | (1) | (1) | (1) |
| Global Rank Focal * Group * Sex Focal | 0.119 | 0.521 | (1) | (1) | (1) |
| Global Rank Partner * Group * Sex Focal | -1.942 | 1.013 | (1) | (1) | (1) |
| Global Rank Partner^2^ * Group * Sex Focal | 0.759 | 0.853 | (1) | (1) | (1) |
| Global Rank Focal * Global Rank Partner * Group | 0.661 | 0.401 | (1) | (1) | (1) |
| Global Rank Focal * Global Rank Partner^2^ * Group | -0.514 | 0.392 | (1) | (1) | (1) |
| Global Rank Focal * Global Rank Partner * Group * Sex Focal | -0.085 | 0.461 | (1) | (1) | (1) |
| **Global Rank Focal * Global Rank Partner** ^2^ *** Group * Sex Focal** | 0.404 | 0.455 | 0.85 | 1 | 0.357 |

1. = not shown because of having a very limited interpretation
2. = reference level is East
3. = reference level is Other
4. = z-transformed, mean and standard deviation in Table S6

**Table S4B: Result of Final Model 2 - Global: Comparing grooming partner choice between chimpanzee males and females, using community-wide rank and relationship variables. Test parameters in bold. Estimate and Standard Error from full model, X², df and p-values from a likelihood ratio test comparing the reduced model lacking this term with the full model. DDSI = Dynamic Dyadic Sociality Index.**

| **Term** | **Estimate** | **SE** | **Χ^2^** | **df** | **p** |
| --- | --- | --- | --- | --- | --- |
| Intercept | -0.439 | 0.336 | (1) | (1) | (1) |
| Group ^(2)^ | -0.409 | 0.239 | (1) | (1) | (1) |
| **Reproductive State Partner Lactating ^(3)^** | 1.039 | 0.196 | 32.51 | 2 | 0.000 |
| **Reproductive State Partner Swelling ^(3)^** | -0.198 | 0.127 |  |  |  |
| Sex Partner (female=0, male=1) | 1.308 | 0.465 | (1) | (1) | (1) |
| Sex Focal (female=0, male=1) | 0.797 | 0.309 | (1) | (1) | (1) |
| Global Rank Focal ^(4)^ | 0.023 | 0.094 | (1) | (1) | (1) |
| Global Rank Partner ^(4)^ | 0.118 | 0.240 | (1) | (1) | (1) |
| Global Rank Partner ^2^ | -0.134 | 0.143 | (1) | (1) | (1) |
| Global DDSI Dyad ^(4)^ | 0.543 | 0.140 | (1) | (1) | (1) |
| **Maximum DDSI Partner ^(4)^** | -0.145 | 0.051 | 6.49 | 1 | 0.011 |
| **Previous Aggression (no=0, yes=1)** | 0.612 | 0.159 | 13.90 | 1 | 0.000 |
| Global Rank Focal * Global Rank Partner | 0.026 | 0.099 | (1) | (1) | (1) |
| Global Rank Focal * Group | -0.046 | 0.106 | (1) | (1) | (1) |
| Global Rank Partner * Group | 0.113 | 0.284 | (1) | (1) | (1) |
| **Global Rank Partner^2^ * Group** | 0.431 | 0.187 | 5.19 | 1 | 0.023 |
| **Global DDSI Dyad * Sex Focal** | -0.393 | 0.148 | 7.09 | 1 | 0.008 |
| **Sex Partner * Sex Focal** | -1.734 | 0.431 | 16.91 | 1 | 0.000 |
| **Global Rank Focal * Global Rank Partner * Group** | 0.363 | 0.135 | 4.48 | 1 | 0.034 |

1. = not shown because of having a very limited interpretation
2. = reference level is East
3. = reference level is Other
4. = z-transformed, mean and standard deviation in Table S6

**Table S5A: Result of Full Model 2 - Relative: Comparing grooming partner choice between chimpanzee males and females, using party-specific rank and relationship variables. Test parameters in bold. Estimate and Standard Error from full model, X², df and p-values from a likelihood ratio test comparing the reduced model lacking this term with the full model. DDSI = Dynamic Dyadic Sociality Index. Full null model comparison: X² = 108.71, df = 22, p < 0.001**

| **Term** | **Estimate** | **SE** | **Χ^2^** | **df** | **p** |
| --- | --- | --- | --- | --- | --- |
| Intercept | -0.334 | 0.396 | (1) | (1) | (1) |
| Group ^(2)^ | 0.453 | 0.415 | (1) | (1) | (1) |
| Reproductive State Partner Lactating ^(3)^ | 0.932 | 0.689 | (1) | (1) | (1) |
| Reproductive State Partner Swelling ^(3)^ | -1.298 | 0.592 | (1) | (1) | (1) |
| Sex Partner (female=0, male=1) | 1.552 | 0.515 | (1) | (1) | (1) |
| Sex Focal (female=0, male=1) | 0.362 | 0.387 | (1) | (1) | (1) |
| Relative Rank Focal ^(4)^ | 0.114 | 0.168 | (1) | (1) | (1) |
| Relative Rank Partner ^(4)^ | 0.592 | 0.286 | (1) | (1) | (1) |
| Relative Rank Partner ^2^ | 0.020 | 0.230 | (1) | (1) | (1) |
| Relative DDSI Dyad ^(4)^ | 0.476 | 0.127 | (1) | (1) | (1) |
| Maximum DDSI Partner ^(4)^ | -0.267 | 0.114 | (1) | (1) | (1) |
| Previous Aggression (no=0, yes=1) | 0.254 | 0.287 | (1) | (1) | (1) |
| Relative Rank Focal * Relative Rank Partner | 0.244 | 0.164 | (1) | (1) | (1) |
| Relative Rank Focal * Relative Rank Partner ^2^ | 0.050 | 0.142 | (1) | (1) | (1) |
| Relative Rank Focal * Sex Focal | 0.068 | 0.188 | (1) | (1) | (1) |
| Relative Rank Partner * Sex Focal | -0.463 | 0.309 | (1) | (1) | (1) |
| Relative Rank Partner^2^ * Sex Focal | 0.044 | 0.233 | (1) | (1) | (1) |
| **Relative DDSI Dyad * Sex Focal** | -0.326 | 0.135 | 5.81 | 1 | 0.016 |
| **Reproductive State Partner Lactating * Sex Focal** | 0.218 | 0.705 | 4.75 | 2 | 0.093 |
| **Reproductive State Partner Swelling * Sex Focal** | 1.155 | 0.604 |  |  |  |
| **Maximum DDSI Partner * Sex Focal** | 0.136 | 0.115 | 2.04 | 1 | 0.154 |
| **Previous Aggression * Group** | 0.603 | 0.356 | 2.41 | 1 | 0.120 |
| Sex Focal * Group | -0.724 | 0.376 | (1) | (1) | (1) |
| Sex Partner * Group | -1.562 | 0.683 | (1) | (1) | (1) |
| Sex Partner * Sex Focal | -1.901 | 0.497 | (1) | (1) | (1) |
| **Sex Partner * Sex Focal * Group** | 2.045 | 0.647 | 11.95 | 1 | 0.001 |
| Relative Rank Focal * Relative Rank Partner * Sex Focal | -0.248 | 0.178 | (1) | (1) | (1) |
| **Relative Rank Focal * Relative Rank Partner ^2^ * Sex Focal** | -0.225 | 0.157 | 2.39 | 1 | 0.122 |

1. = not shown because of having a very limited interpretation
2. = reference level is East
3. = reference level is Other
4. = z-transformed, mean and standard deviation in Table S6

**Table S5B: Result of Final Model 2 - Relative: Comparing grooming partner choice between chimpanzee males and females, using party-specific rank and relationship variables. Test parameters in bold. Estimate and Standard Error from full model, X², df and p-values from a likelihood ratio test comparing the reduced model lacking this term with the full model. DDSI = Dynamic Dyadic Sociality Index.**

| **Term** | **Estimate** | **SE** | **Χ^2^** | **df** | **p** |
| --- | --- | --- | --- | --- | --- |
| Intercept | -0.410 | 0.314 | (1) | (1) | (1) |
| Group ^(2)^ | 0.450 | 0.410 | (1) | (1) | (1) |
| **Reproductive State Partner Lactating ^(3)^** | 1.112 | 0.203 | 33.87 | 2 | 0.000 |
| **Reproductive State Partner Swelling ^(3)^** | -0.204 | 0.133 |  |  |  |
| Sex Partner (female=0, male=1) | 1.443 | 0.499 | (1) | (1) | (1) |
| Sex Focal (female=0, male=1) | 0.490 | 0.301 | (1) | (1) | (1) |
| Relative Rank Focal ^(4)^ | 0.149 | 0.121 | (1) | (1) | (1) |
| Relative Rank Partner ^(4)^ | 0.595 | 0.262 | (1) | (1) | (1) |
| Relative DDSI Dyad ^(4)^ | 0.433 | 0.126 | (1) | (1) | (1) |
| **Maximum DDSI Partner ^(4)^** | -0.159 | 0.053 | 6.73 | 1 | 0.009 |
| **Previous Aggression (no=0, yes=1)** | 0.651 | 0.168 | 13.24 | 1 | 0.000 |
| **Relative DDSI Dyad * Sex Focal** | -0.291 | 0.134 | 5.45 | 1 | 0.019 |
| Relative Rank Focal * Relative Rank Partner | 0.247 | 0.146 | (1) | (1) | (1) |
| Relative Rank Focal * Sex Focal | -0.106 | 0.137 | (1) | (1) | (1) |
| Relative Rank Partner * Sex Focal | -0.399 | 0.284 | (1) | (1) | (1) |
| Sex Focal * Group | -1.837 | 0.487 | (1) | (1) | (1) |
| Sex Partner * Group | -0.641 | 0.374 | (1) | (1) | (1) |
| Sex Partner * Sex Focal | -1.381 | 0.673 | (1) | (1) | (1) |
| **Sex Partner * Sex Focal * Group** | 1.794 | 0.644 | 7.71 | 1 | 0.006 |
| **Relative Rank Focal * Relative Rank Partner * Sex Focal** | -0.291 | 0.161 | 3.45 | 1 | 0.063 |

1. = not shown because of having a very limited interpretation
2. = reference level is East
3. = reference level is Other
4. = z-transformed, mean and standard deviation in Table S6

**Table S6: Original means and standard deviations of z-transformed variables for all models (Global = in comparison to whole community, Relative = in comparison to party, DDSI = Dynamic Dyadic Sociality Index)**

| **Model** | **Variable** | **Mean** | **SD** |
| --- | --- | --- | --- |
| 1 | Global Rank Focal | 0.795 | 0.220 |
|  | Global Rank Partner | 0.654 | 0.253 |
|  | Relative Rank Focal | 0.707 | 0.246 |
|  | Relative Rank Partner | 0.551 | 0.287 |
|  | Global DDSI Dyad | 0.526 | 0.118 |
|  | Relative DDSI Dyad | 0.587 | 0.287 |
|  | Maximum DDSI Partner | 0.634 | 0.112 |
| 2 | Global Rank Focal | 0.845 | 0.163 |
|  | Global Rank Partner | 0.673 | 0.246 |
|  | Relative Rank Focal | 0.740 | 0.226 |
|  | Relative Rank Partner | 0.547 | 0.286 |
|  | Global DDSI Dyad | 0.527 | 0.123 |
|  | Relative DDSI Dyad | 0.588 | 0.287 |
|  | Maximum DDSI Partner | 0.642 | 0.114 |
